# Rheumatoid Arthritis Prevalence and Risk Factors in Korean Adults: A Focus on Age and Sex Differences

**DOI:** 10.3390/medsci13010017

**Published:** 2025-02-09

**Authors:** Do-Youn Lee

**Affiliations:** College of General Education, Kookmin University, Seoul 02707, Republic of Korea; triptoyoun@kookmin.ac.kr; Tel.: +82-02-910-5540

**Keywords:** rheumatoid arthritis, prevalence, risk factors

## Abstract

Rheumatoid arthritis (RA) is a chronic autoimmune disorder that affects the joints, causing swelling, pain, stiffness, and functional decline. This study aims to clarify the prevalence and risk factors of RA based on sex and age among Korean adults, providing essential data for targeted prevention and management strategies. We analyzed data from the Korea National Health and Nutrition Examination Survey 2016–2021, comprising 25,166 participants aged 20 and older. Sociodemographics, health status, and behavior factors were evaluated, with RA defined based on self-reported diagnosis. A complex sampling design was utilized to ensure representative results and multiple logistic regression was employed to determine the risk factors linked to RA. The overall prevalence of RA among Korean adults was 1.1%, showing a significant sex-based disparity: 0.6% and 1.7% in men and women, respectively. RA prevalence increased with age, peaking at 3.5% in individuals over 70. This study identified education level, subjective health status, and age as key predictors of RA. Among men, significant predictors of RA included education level and subjective health status, with a higher risk observed in men with only elementary education and poor perceived health. For women, age and subjective health status were the main risk factors, with RA risk increasing markedly in older age groups, particularly in those aged 70 and above. This study highlights the distinct prevalence and risk factors for RA among Korean adults by sex and age. Key predictors—education level, subjective health status, and age—suggest that tailored health interventions addressing these factors are crucial to reducing the RA burden and enhancing health outcomes among affected populations.

## 1. Introduction

Rheumatoid arthritis (RA) is a chronic autoimmune disease marked by systemic inflammation that predominantly targets the joints, causing pain, swelling, stiffness, and substantial functional impairment [1]. The clinical progression of RA varies widely among patients; while some experience milder symptoms, chronic RA often leads to severe joint deformities and disabilities, negatively affecting quality of life. Without appropriate management, RA can cause long-term physical damage and increase the risk of premature mortality, highlighting the need for timely diagnosis and continuous care [2,3]. Consequently, RA is recognized as not only a joint-related condition but also a major public health issue with substantial social and economic effects on individuals and healthcare systems [4,5].

To understand disease burden and trends, periodically studying prevalence across different countries is essential [4,6]. The prevalence of rheumatoid arthritis (RA) varies based on population characteristics and regions, but it is reported that women have a prevalence rate more than twice that of men [7,8]. This sex difference likely stems from various biological factors, including genetics and hormonal changes [9,10]. Although countries in Asia, including South Korea, have relatively lower prevalence rates than those of Western countries, incidence is rising due to lifestyle changes and an aging population [8,11]. Most RA cases occur in adulthood, with high prevalence observed in middle-aged individuals; however, the incidence is also gradually increasing among the elderly [12]. Therefore, research into these prevalence trends is critical for a precise understanding of RA and its risk factors. Additionally, a study on RA prevalence and associated factors is crucial for informing public health priorities and resource allocation [4].

Previous studies show that genetic, environmental, and lifestyle factors collectively act as risk factors for RA [9,10,13,14]. Hormonal changes, smoking, and exposure to specific infections are particularly recognized as major contributors to an increased risk of developing RA [14,15]. Metabolic factors, such as obesity, along with stress and psychological factors, are also gaining attention as associated factors [16,17]. These diverse risk factors are crucial considerations in managing and preventing RA. Therefore, a comprehensive understanding of RA prevalence and risk factors is essential for establishing effective prevention strategies and tailoring treatments to individual patient characteristics.

This study aims to comprehensively analyze the prevalence and risk factors of RA based on sex and age to better clarify the disease characteristics in South Korea. The specific objectives are as follows: (1) to determine the prevalence of RA by sex and age group, (2) to analyze the sociodemographic and health-related characteristics of RA by sex, and (3) to identify sex-specific risk factors for RA.

## 2. Materials and Methods

Data from the Korea National Health and Nutrition Examination Survey (KNHANES), conducted between 2016 and 2021, were used in this study. KNHANES is a large-scale, ongoing national survey that provides extensive information on the health and nutritional status of the Korean population, making it a valuable resource for population-level health studies.

**Figure 1:**
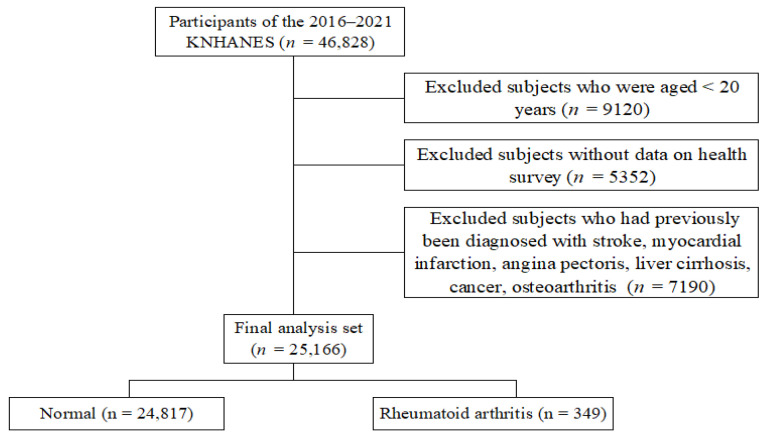
Selection process of participants from the Korea National Health and Nutrition Examination Survey 2016–2021.

In total, 46,828 participants were initially included in the KNHANES dataset. However, exclusion criteria were applied to refine the sample and enhance analysis accuracy by minimizing confounding factors. First, individuals under the age of 20 were excluded, removing 9120 participants from the dataset to focus on adults whose lifestyle and health factors may more directly relate to RA prevalence. Additionally, 5352 participants with incomplete health screenings or survey components were excluded, as incomplete information could compromise the reliability and validity of the findings. Furthermore, 7190 individuals with severe health conditions, such as stroke, myocardial infarction, or angina, were removed to reduce confounding effects from significant comorbidities that may independently influence RA prevalence and related health outcomes. Finally, 25,166 participants met the inclusion criteria, and they were selected for the study (Figure 1).

### 2.1. Sociodemographic Factors

This analysis considered sociodemographic factors, including sex, age, educational level, marital status, and personal income level. Sex was categorized as male or female. Age groups were categorized into 20s, 30s, 40s, 50s, 60s, and 70s or older. Educational level was categorized based on completion of elementary school, middle school, high school, and university. Marital status was based on whether participants were currently living with a spouse. Personal income level was divided into quartiles to categorize average monthly income.

### 2.2. Health Status Factors

This study evaluated various health and disease-related factors, including height, weight, body mass index (BMI), blood pressure, fasting glucose, triglyceride levels, high-density lipoprotein cholesterol (HDL-C), waist circumference (WC), and smoking and drinking habits, as well as aerobic and resistance exercise patterns. BMI was calculated by dividing weight in kilograms by height in meters squared (kg/m^2^) and was categorized into underweight, normal weight, overweight, and obese groups. Hypertension was defined as a systolic blood pressure above 130 mmHg, diastolic pressure above 85 mmHg, or current use of antihypertensive medications. Diabetes was identified via a fasting glucose level of ≥100 mg/dL or current use of diabetes medication. High triglycerides were defined as levels > 150 mg/dL. Low HDL-C was specified as <40 mg/dL for men and <50 mg/dL for women, while abdominal obesity was classified as a WC above 90 cm for men and over 85 cm for women.

### 2.3. Health Behavior Factors

Smoking status was categorized by classifying individuals who reported “daily smoking” or “occasional smoking” as current smokers, those who indicated “I smoked in the past but do not smoke now” as past smokers, and those who responded “I never smoked” as nonsmokers. Current drinking was defined as consuming alcohol “at least once a month”, while nondrinking included those who drank “less than once a month” or “did not drink at all in the past year”.

Aerobic exercise was assessed based on walking duration, with participants reporting the number of days they walked for at least 10 min over the past week. An aerobic exercise rate was established for those who accumulated 150 min or more of walking per week. Resistance exercise frequency was determined by asking participants how many times per week they engaged in resistance activities, such as push-ups, sit-ups, or lifting weights. Resistance exercise participants were classified into the following groups: those who ‘never’ engaged in resistance activities, individuals who exercised 1–3 days per week (moderate intensity), and those who exercised more than 4 days per week (high intensity).

Subjective health status was classified based on the following survey responses: “Very Good” or “Good” were categorized as “Good”, “Moderate” were categorized as “Normal”, and “Poor” or “Very Poor” were classified as “Bad”.

### 2.4. Rheumatoid Arthritis

In this study, participants were identified as having RA based on their responses to a survey question, “Are you currently suffering from RA?” The RA condition was categorized as “Yes” or “No”. Participants who answered “Yes” were identified as RA cases, indicating a current diagnosis or experience of the condition. Conversely, those who answered “No” were categorized as non-RA cases, signifying they were not currently experiencing symptoms, or they had not been diagnosed with RA.

### 2.5. Data Analysis

Statistical analyses were performed using SPSS version 28.0, with a significance level set at 0.05. To examine RA prevalence and associated risk factors within a nationally representative sample, a complex sampling approach was applied to the KNHANES data, ensuring the findings accurately reflect the Korean adult population. This approach incorporated survey weights, stratification, and clustering to adjust for the stratified sampling of the multistage, accounting for sampling errors and design effects, which can improve the reliability and generalizability of the results.

The analysis methods included the following: First, differences in characteristics between the RA and normal groups were examined using a complex sample χ^2^-test, with variance estimation based on standard errors. Second, complex sample multiple logistic regression was employed to identify risk factors related to RA, with results presented as odds ratios (OR) and 95% confidence intervals (CI).

## 3. Results

### 3.1. Prevalence of Rheumatoid Arthritis Based on Sex and Age Groups

**Figure 2:**
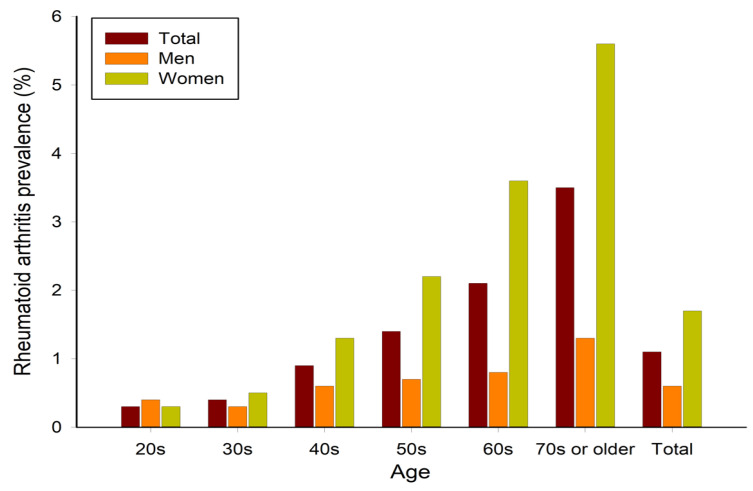
Prevalence of rheumatoid arthritis in Korea.

Table 1 and Figure 2 present the prevalence of RA by sex and age groups. Among South Korean adults aged 20 or older, the overall RA prevalence was 1.1%, with a significant difference observed between the following sexes: 0.6% in males and 1.7% in females. The prevalence of RA was lowest among individuals in their 20s and increased with age, peaking at 3.5% in the 70s. This trend highlights the significance of monitoring RA in older populations, where the risk is higher.

### 3.2. Sociodemographic Characteristics of Study Participants

Table 2 shows the sociodemographic characteristics of study participants based on sex and RA. Among men, significant differences were observed in educational levels, with higher RA prevalence among those with lower education. In contrast, significant differences were observed in age groups and educational levels among women, suggesting that these factors may play a pivotal role in RA development and management.

### 3.3. Health Status Characteristics of Study Participants

Table 3 outlines the health status characteristics of study participants based on sex and RA. Among males, significant differences were observed only in subjective health status. In women, significant differences were observed in drinking status, subjective health status, and hypertension.

### 3.4. Risk Factors for Rheumatoid Arthritis

Table 4 and Table 5 detail the factors influencing the RA occurrence based on sex. Logistic regression analysis revealed significant differences in risk factors for RA among men, particularly regarding education level and subjective health status. Men with only an elementary school education had approximately 3.44 times higher risk of developing RA than that of college graduates, underscoring the critical role of educational attainment in health outcomes. Additionally, men with a subjective health status classified as “Bad” had a 3.79 times higher risk of developing RA than those rated as “Good”, indicating an association between perceived health and disease prevalence.

For women, age group and subjective health status were identified as significant RA risk factors. Compared to women in their 20s, the likelihood of developing RA was approximately 5.00, 8.43, 13.30, and 18.82 times higher in those in their 40s, 50s, 60s, and 70s, respectively, with no significant difference observed in their 30s. Furthermore, women with a subjective health status rated as ‘Bad’ had a 5.40 times higher risk of RA than those rated as “Good”, while those rated as “Normal” had a 2.33 times higher risk. These findings highlight the significance of monitoring subjective health perceptions and educational background in RA management and prevention.

## 4. Discussion

This study was conducted to identify the prevalence and risk factors of RA based on sex and age, aiming to offer effective prevention strategies and health management for the disease. The results showed that RA prevalence among South Korean adults was 1.1%, with 0.6% and 1.7% in men and women, respectively. The lowest prevalence was observed in individuals in their 20s, with a trend of increasing prevalence with age. Women showed a higher prevalence than men, particularly pronounced in their 70s.

RA currently impacts approximately 17.6 million people worldwide, with projections indicating an increase to 31.7 million by 2050. Furthermore, women comprise approximately two-thirds of these patients, showing significantly higher rates than men across all age groups [17]. These findings align with the results of this study, indicating a pattern of increased RA prevalence among women. This difference could be attributed to hormonal, immunological, genetic, and environmental attributes. Increased risk could also be due to estrogen fluctuations [18], increased immune activity [9], and genetic variants [9,10,13]. Moreover, environmental factors, such as smoking and lifestyle differences, may additionally affect RA risk among women.

The findings of this study indicate that education level and subjective health status are significant risk factors for RA in men. Men with a lower education level, such as those with only elementary school education, exhibited a significantly higher risk of developing RA than their counterparts with college degrees, as evidenced by an OR of 3.435 (95% CI 1.682–7.015). This finding aligns with previous studies that report an inverse relationship between education level and RA prevalence [19,20,21,22,23]. One study suggests that low education levels are related to lower income and physically demanding jobs, which may predict increased RA prevalence [22]. Another study shows that individuals with higher socioeconomic status and over 12 years of education have a significantly reduced risk of RA-related hospitalization, likely due to lower exposure to stressors and nutritional deficiencies than those with lower socioeconomic status [19]. Additionally, another study suggests that individuals with lower educational levels tend to exhibit higher arthritis prevalence, potentially due to preceding risk factors such as high-risk jobs, obesity, and greater joint damage [21]. These findings underscore the significance of implementing RA health management education programs targeting men with lower education levels and enhancing support for early diagnosis and treatment to improve disease outcomes.

This study reveals a trend indicating that the risk of developing RA in women rises with age. Although no significant difference was observed between women in their 20s and 30s, the OR for older age groups was significantly elevated: OR 4.995 (95% CI 1.682–14.833) for the 40s, OR 8.335 (95% CI 2.822–24.62) for the 50s, OR 13.298 (95% CI 4.441–39.819) for the 60s, and OR 18.822 (95% CI 6.116–57.927) for the 70s. These findings highlight a concerning trend of increasing RA risk with advancing age in women. A study also reports that RA prevalence peaked in the 75–79 age group in 2020, confirming the age-related nature of this disease [18]. Furthermore, research shows that RA incidence rates increase with age, with women and men exhibiting the highest prevalence in their 70s [24]. Moreover, a study conducted in Japan highlights a demographic shift, with the peak age for RA onset moving from 50–59 years in 2002 to 60–69 years in 2012, revealing that RA prevalence in the 60s, 70s, and 80s increased significantly by 118%, 153%, and 251%, respectively, during this period. Conversely, the prevalence in the 30s declined significantly by 58.6% over the same decade [25]. These findings suggest that RA incidence is indeed age-related, emphasizing the critical need for age-specific prevention and management strategies, as tailored interventions may improve health outcomes across different age groups.

Subjective health status emerged as a significant risk factor for RA in both men and women. Compared to those who responded ‘Good’, men who reported ‘Bad’ health had an OR of 3.790 (95% CI 1.867–7.696), indicating a substantial risk increase associated with a poor self-assessment of health. In women, those who responded ‘Normal’ showed an OR of 2.328 (95% CI 1.48–3.661), while those reporting ‘Bad’ health exhibited an even higher OR of 5.401 (95% CI 3.321–8.784). These findings align with those of the existing literature, suggesting that the subjective health status in RA patients can significantly influence disease progression [26]. Subjective health status is closely linked to various factors, such as the ability of a patient to manage illness, adherence to treatment, and social support from family and friends [26]. These aspects underscore the need to incorporate subjective health evaluations into RA treatment strategies, as they offer valuable insights into how patients perceive and experience their condition. Furthermore, prior research shows that RA-associated pain negatively influences overall subjective health and satisfaction, correlating significantly with psychological states, such as tension and mood [27]. These findings underscore the notion that the health perception of a patient can directly affect their physical and psychological resilience. Therefore, evaluating subjective health status and objective indicators is essential for the comprehensive approach to RA treatment.

This study has some limitations. First, the criteria used to define RA relied on self-reported surveys, which do not capture pain severity or other clinical measures. Second, as a cross-sectional study, this research examined RA prevalence and associated risk factors at a single point in time using secondary data. This design limits the ability to establish causal relationships between identified risk factors and RA development, as it does not allow for tracking changes over time. Furthermore, strategies to address the gaps in future research, such as integrating clinical data or conducting longitudinal studies to establish causal relationships, have not been sufficiently proposed. To address these limitations, future studies with a cohort design are needed to explore these causal relationships more thoroughly and provide a deeper understanding of the underlying dynamics. Third, the exclusion of some severe RA cases from the KNHANES survey may have influenced outcome analysis. However, since the KNHANES data represent a nationwide population, the effect of nonparticipating individuals on the study results is limited. Fourth, the dataset used in this study did not provide variables related to hormonal or genetic factors. Therefore, future research should include investigations into these aspects to offer a more comprehensive understanding. Fifth, psychological stress was not directly included in the following model but was used in subjective health status and comorbidities, which may indirectly reflect psychological status. Future studies would ideally include direct stress measurement, as this would allow a clearer estimation of its role in RA.

Considering these limitations, this study emphasizes the importance of proposing adaptable health management strategies rather than narrowly customized interventions. Specifically, the findings highlight the need to provide policymakers and public health authorities with a flexible framework that can be adjusted based on demographic and health-specific characteristics. Such an approach is designed to effectively tackle RA prevalence and curb disease progression, taking into account the diversity in data and the unique needs of various populations.

## 5. Conclusions

In this study, we analyzed the prevalence and risk factors of RA among Korean adults by sex and age, offering insights into the characteristics of the disease and providing foundational data for prevention and management strategies. The findings indicate an overall RA prevalence of 1.1% among adults aged 20 and older, with rates of 0.6% in men and 1.7% in women. Common risk factors for RA include subjective health status for both sexes, with educational level and older age identified as risk factors for men and women, respectively. Therefore, it is crucial to develop flexible health management strategies that can be adapted to the demographic and health-specific characteristics of different populations in order to reduce RA prevalence and prevent disease progression.

## Figures and Tables

**Table 1:** Prevalence of rheumatoid arthritis based on sex and age groups.

Prevalence	20s	30s	40s	50s	60s	70s	Total
Total	0.3	0.4	0.9	1.4	2.1	3.5	1.1
Men	0.4	0.3	0.6	0.7	0.8	1.3	0.6
Women	0.3	0.5	1.3	2.2	3.6	5.6	1.7

**Table 2:** Sociodemographic characteristics of participants with rheumatoid arthritis based on sex.

Factors	Categories	Men			Women		
		RA (*n* = 83)	Normal (*n* = 11,645)	*p*	RA (*n* = 266)	Normal (*n* = 13,172)	*p*
		M or %	M or %		M or %	M or %	
Age	20s	13.5	19.6	0.097	3.1	19.4	<0.001
	30s	11.2	20.6		5.8	20.5	
	40s	21.7	22.5		17.9	23.0	
	50s	24.1	19.8		27.2	20.2	
	60s	16.0	11.6		23.6	10.6	
	70s or older	13.5	5.9		22.4	6.3	
Education	Elementary	14.4	4.9	0.001	27.9	9.5	<0.001
	Middle	6.7	6.2		11.9	7.0	
	High	35.0	27.6		30.0	29.1	
	University	43.9	61.4		30.2	54.5	
Marital status	With	66.2	64.7	0.833	71.8	65.8	0.076
	Without	33.8	35.3		28.2	34.2	
Personal income	Q1 (Lowest)	22.5	24.1	0.094	23.7	24.1	0.981
	Q2	39.1	25.1		26.3	25.1	
	Q3	19.3	25.5		24.8	25.4	
	Q4 (Highest)	19.1	25.3		25.2	25.4	

RA, rheumatoid arthritis; Q, quartiles.

**Table 3:** Health status and behavior characteristics of participants with rheumatoid arthritis based on sex.

Factors	Categories	Men			Women		
		RA (*n* = 83)	Normal (*n* = 11,645)	*p*	RA (*n* = 266)	Normal (*n* = 13,172)	*p*
		M or %	M or %		M or %	M or %	
BMI	Low	5.0	2.2	0.346	5.9	6.2	0.616
	Normal	49.9	53.7		68.3	67.8	
	Overweight	41.0	36.9		22.5	20.9	
	Obesity	4.1	7.2		3.3	5.1	
Smoking status	Current	38.7	36.2	0.302	4.8	6.3	0.171
	Past	43.3	37.2		4.4	7.2	
	Non	18.0	26.5		90.8	86.4	
Drinking status	Yes	63.7	72.8	0.125	32.2	48.8	<0.001
	No	36.3	27.2		67.8	51.2	
Aerobic exercise	Yes	47.8	50.4	0.700	55.3	52.6	0.434
Resistance exercise	Never	73.2	64.4	0.064	82.5	80.7	0.523
	Mid	20.9	19.4		10.6	13.3	
	High	5.9	16.1		6.9	6	
Subjective health status	Bad	29.3	12.5	<0.001	37.9	15.0	<0.001
	Normal	51.0	51.2		50.4	53.3	
	Good	19.7	36.3		11.7	31.7	
Comorbidities conditions							
Hypertension		43.1	33.7	0.138	31.6	19.0	<0.001
Diabetes		40.3	40.3	0.991	30.3	25.7	0.127
High triglyceride		32.1	39.3	0.238	20.2	18.0	0.422
Low HDL-C		26.4	26.1	0.954	39.1	34.0	0.118
Abdominal obesity		45.4	36.5	0.174	26.2	22.9	0.205

RA, rheumatoid arthritis; HDL-C, high-density lipoprotein-cholesterol.

**Table 4:** Multiple logistic regression for rheumatoid arthritis risk factors in men.

Factors	Categories	Crude		Adjusted	
		OR (95% CI)	*p*	OR (95% CI)	*p*
Education	Elementary	4.153 (2.062–8.364)	<0.001	3.435 (1.682–7.015)	<0.001
	Middle	1.533 (0.629–3.741)	0.348	1.315 (0.529–3.27)	0.557
	High	1.773 (0.936–3.361)	0.079	1.658 (0.87–3.162)	0.125
	University	1		1	
Subjective health status	Bad	4.328 (2.174–8.616)	<0.001	3.790 (1.867–7.696)	<0.001
	Normal	1.839 (0.95–3.563)	0.071	1.719 (0.888–3.328)	0.108
	Good	1		1	

**Table 5:** Multiple logistic regression for rheumatoid arthritis risk factors in women.

Factors	Categories	Crude		Adjusted	
		OR (95% CI)	*p*	OR (95% CI)	*p*
Age	20s	1		1	
	30s	1.812 (0.547–6.004)	0.331	1.814 (0.55–5.984)	0.328
	40s	4.950 (1.651–14.844)	0.004	4.995 (1.682–14.833)	0.004
	50s	8.535 (2.873–25.358)	<0.001	8.335 (2.822–24.62)	<0.001
	60s	14.132 (4.787–41.721)	<0.001	13.298 (4.441–39.819)	<0.001
	70s or older	22.412 (7.489–67.07)	<0.001	18.822 (6.116–57.927)	<0.001
Education	Elementary	5.314 (3.69–7.655)	<0.001	0.962 (0.598–1.549)	0.873
	Middle	3.097 (1.984–4.836)	<0.001	0.833 (0.507–1.368)	0.469
	High	1.859 (1.278–2.704)	0.001	0.92 (0.627–1.351)	0.67
	University	1		1	
Alcohol status	Yes	0.498 (0.371–0.668)	<0.001	0.888 (0.65–1.214)	0.455
	No	1		1	
Subjective health status	Bad	6.865 (4.276–11.023)	<0.001	5.401 (3.321–8.784)	<0.001
	Normal	2.558 (1.627–4.024)	<0.001	2.328 (1.48–3.661)	<0.001
	Good	1		1	
Blood pressure	Normal	1		1	
	Hypertension	1.970 (1.469–2.641)	<0.001	0.917 (0.678–1.241)	0.574

## Data Availability

All the data were anonymized and are available for download from the website (https://knhanes.kdca.go.kr/knhanes, accessed on 10 October 2024).
